# Using pCO_2_ Gap in the Differential Diagnosis of Hyperlactatemia Outside the Context of Sepsis: A Physiological Review and Case Series

**DOI:** 10.1155/2019/5364503

**Published:** 2019-12-04

**Authors:** Petr Waldauf, Katerina Jiroutkova, Frantisek Duska

**Affiliations:** ^1^Department of Anaesthesia and Intensive Care Medicine, The Third Faculty of Medicine, Charles University and FNKV University Hospital, Prague, Czech Republic; ^2^Oxylab: Lab of Mitochondrial Physiology, The Third Faculty of Medicine, Charles University, Prague, Czech Republic

## Abstract

**Introduction:**

There is an inverse relationship between cardiac output and the central venous-arterial difference of partial pressures of carbon dioxide (pCO_2_ gap), and pCO_2_ gap has been used to guide early resuscitation of septic shock. It can be hypothesized that pCO_2_ gap can be used outside the context of sepsis to distinguish type A and type B lactic acidosis and thereby avoid unnecessary fluid resuscitation in patients with high lactate, but without organ hypoperfusion.

**Methods:**

We performed a structured review of the literature enlightening the physiological background. Next, we retrospectively selected a series of case reports of nonseptic critically ill patients with elevated lactate, in whom both arterial and central venous blood gases were simultaneously measured and the diagnosis of either type A or type B hyperlactataemia was conclusively known. In these cases, we calculated venous-arterial CO_2_ and O_2_ content differences and pCO_2_ gap.

**Results:**

Based on available physiological data, pCO_2_ can be considered as an acceptable surrogate of venous-arterial CO_2_ content difference, and it should better reflect cardiac output than central venous saturation or indices based on venous-arterial O_2_ content difference. In our case report of nonseptic patients, we observed that if global hypoperfusion was present (i.e., in type A lactic acidosis), pCO_2_ gap was elevated (>1 kPa), whilst in the absence of it (i.e., in type B lactic acidosis), pCO_2_ gap was low (<0.5 kPa).

**Conclusion:**

Physiological rationale and a small case series are consistent with the hypothesis that low pCO_2_ gap in nonseptic critically ill is suggestive of the absence of tissue hypoperfusion, mandating the search for the cause of type B lactic acidosis rather than administration of fluids or other drugs aimed at increasing cardiac output.

## 1. Introduction

Differential diagnosis of elevated blood lactate is the daily bread and butter of all clinicians looking after critically ill patients. In principle, hyperlactatemia can either be caused by increased production in tissues (type A) or impaired lactate uptake (type B) or by these two mechanisms combined [1, 2]. Correctly determining the cause(s) of hyperlactatemia is of utmost importance, indeed, because it determines the treatment, which can be life saving for a patient with one underlying cause, but harmful for another. A classical example of this concept is fluid resuscitation that can be helpful in correcting tissue hypoperfusion, but harmful for patients with other causes of elevated lactate. Nice as it sounds, recognizing patients that would benefit from fluid administration (or other ways of increasing cardiac output) is often very difficult at the bedside and often results in fluid abuse, particularly in patients with elevated lactate and on vasopressors [3]. It was recognized in sepsis that pCO_2_ gap (or its mathematical derivatives) outperformed other markers in detecting tissue hypoperfusion [4–7]. We hypothesize that the difference in carbon dioxide partial pressure between central venous and arterial blood (pCO_2_ gap) can be a useful aid in the differential diagnosis of elevated lactate also outside the context of sepsis. In this paper, we present the theoretical rationale for this hypothesis and review published data and confront them with observations of pCO_2_ gaps in a case series of nonseptic patients with known cause of lactate elevation.

### 1.1. Physiological Background

During the process of cellular respiration, carbon dioxide is produced mostly by decarboxylation reactions in citric acid cycle and diffuses into the bloodstream through the extracellular fluid. Whilst approximately 10% of the carbon dioxide (CO_2_) in the blood remains dissolved in the plasma, the remaining CO_2_ diffuses rapidly into the red blood cells, where it is either bound to terminal NH_2_ groups (30%) forming carbaminohaemoglobin or reacts with water to form carbonic acid (60%) that dissociates to bicarbonate and a proton (H^+^) [8]:

(1) $$\begin{array}{c} {\text{CO}}_{2}+{\text{H}}_{2}\text{O}⟷{\text{H}}_{2}{\text{CO}}_{3}⟷{\text{H}}^{+}+{{\text{HCO}}_{3}}^{-} \end{array}$$

The production of HCO_3_^−^ occurs rapidly because of catalysis by carbonic anhydrase. The HCO_3_^−^ then leaves the red blood cells in exchange of Cl^−^ (the process known as chloride or Hamburger shift), thereby promoting the entry of more CO_2_ into the red blood cell. In the bloodstream, CO_2_ in all 3 forms is conveyed back to the respiratory surfaces at a rate, which is directly proportional to cardiac output.

Cardiac output (CO) calculated using the Fick principle applied to CO_2_ agrees well with cardiac output calculated from O_2_-derived parameters in normal subjects at rest and during exercise [9]. The Fick equation (for indirect CO calculation) applied to CO_2_ is

(2) $$\begin{array}{c} \text{CO}=\frac{{\text{VCO}}_{2}}{{\text{VctCO}}_{2}\left(\text{B}\right)-{\text{ActCO}}_{2}\left(\text{B}\right)}, \end{array}$$

where VCO_2_ is the CO_2_ production, CO is the cardiac output, and VctCO_2_ − ActCO_2_ is the venous-to-arterial CO_2_ content difference. After substituting ctCO_2_ gap into the abovementioned equation, we obtain

(3) $$\begin{array}{c} {\text{ctCO}}_{2}\left(\text{B}\right)\text{gap}=\frac{{\text{VCO}}_{2}}{\text{CO}}, \end{array}$$

where the ctCO_2_ (B) gap is inversely related to CO and proportional to VCO_2._ The value for ctCO_2_ is not directly measured; instead, it is calculated from measured pH and pCO_2_, which is a mathematically complex and error-prone process, whereas pCO_2_ is a directly measured parameter that is readily available to clinicians. In this paper, we will determine the pCO_2_ gap by subtracting the peripheral arterial pCO_2_ from the central venous pCO_2_. This is because central venous pCO_2_ has been shown to be a reliable substitute for mixed-venous pCO_2_ [10], and pulmonary artery is rarely catheterized in contemporary ICU practice because of the invasiveness of the procedure [11].

**Figure 1:**
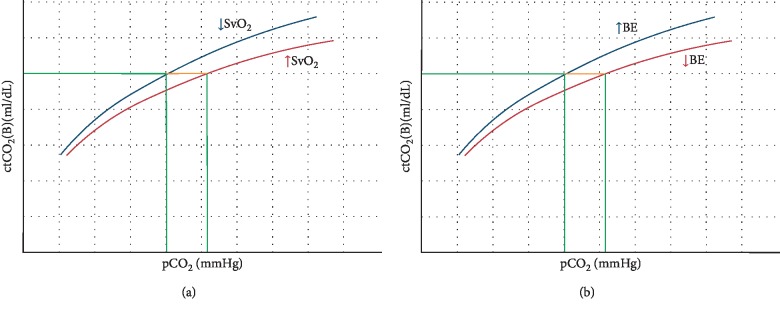
The relationship between partial pressure of CO_2_ (pCO_2_) and whole-blood CO_2_ content (ctCO_2_) and the influence of saturation of venous blood with oxygen (SvO_2_, left) (a) and base excess (BE, right) (b), respectively.

Over the physiologic range of pCO_2,_ the relationship between pCO_2_ and the total blood CO_2_ content is close to linear, so pCO_2_ may be considered a reliable substitute for CO_2_ content [12, 13]. Factors that disturb the linearity between pCO_2_ and CTCO_2_ tend to offset each other for a given CtCO_2_, and pCO_2_ is higher in metabolic acidosis and lower for lower saturation of haemoglobin with oxygen (see Figure 1). During low flow states, organic acids (mainly lactate) are released from hypoperfused tissues, causing base excess of venous blood to be more negative as compared with the arterial blood. On the other hand, venous blood leaving the hypoperfused tissue tend to be more deoxygenated, causing pCO_2_ to be lower for a given CtCO_2_ (Haldane effect [14]). In turn, it can be hypothesized that A-V pCO_2_ gap is representative of CTCO_2_ gap under a wide range of clinical situations.

**Figure 2:**
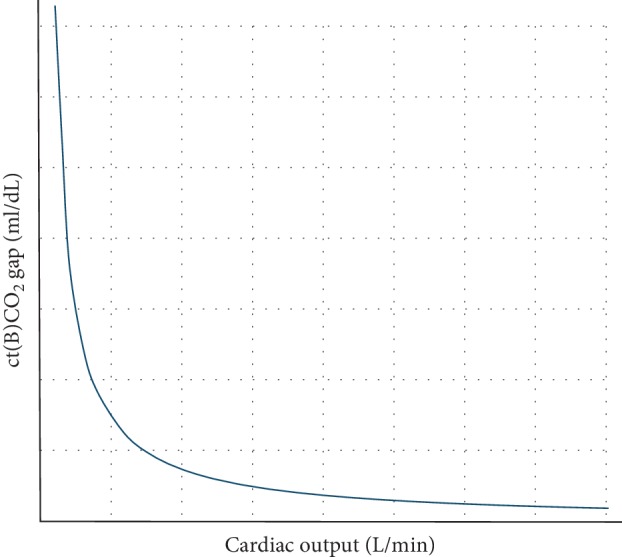
Relationship between cardiac output and central venous-to-arterial difference of CO_2_ content in blood (ct(B)CO_2_ gap).

In turn, when pCO_2_ replaces CtCO_2_ in equation (3), we get

(4) $$\begin{array}{c} \left({\text{PvCO}}_{2}\;–\;{\text{PaCO}}_{2}\right)={\text{pCO}}_{2}\left(\text{B}\right)\text{gap}=\frac{{\text{VCO}}_{2}}{\text{CO}}*k, \end{array}$$

where (PvCO_2_ − PaCO_2_) is the venous-to-arterial PCO_2_ difference and *k* is the PCO_2_ to CTCO_2_ correlation (assumed to be constant). In line, both CTCO_2_ and pCO_2_ gaps were found to increase with the decrease in CO, and the relationship follows a hyperbolic pattern (see Figure 2) [15, 16].

Moreover, elevated CO_2_ gap can be considered as the marker of the cardiac output in relation of peripheral metabolic requirements. Hypoxic hypoxia in the presence of adequate cardiac output cannot cause pCO_2_ gap elevation as demonstrated by elegant experiments of Vallet et al. [17]. On the other hand, if there is an inhomogeneity of distribution of perfusion as it is the case in sepsis, the pCO_2_ gap is more sensitive than ScvO_2_ drop in detecting patients who would benefit from measures aimed at increasing cardiac output [4–6, 18–20]. The cut-off value of pCO_2_ gap in a septic patient was found to be 0.8 kPa (6 mmHg) [20, 21]. The superiority of pCO_2_ gap over ScvO_2_ desaturation in detecting hypoperfusion likely reflects the fact that in the presence of microvascular shunting, central venous blood is a mixture of arterialized blood from shunts and desaturated blood from the hypoperfused regions. Because of 20 times higher diffusibility of CO_2_ as compared with O_2_ [22, 23] central venous blood can have normal or high ScvO_2_ (due to arterialized blood form shunts), whilst CO_2_ content is elevated proportionally to the degree of peripheral tissue hypoperfusion as shunting capillaries are still capable to drain CO_2_ from the hypoperfused tissues. It has been proposed (L. Gattinoni–personal communication) that local tissue acidosis caused by hypoperfusion releases free CO_2_ from bicarbonate, thereby increasing venous pCO_2_ even further, a phenomenon called “Coca Cola effect” in analogy to releasing bubbles by adding piece of lemon into a carbonated drink. In addition, some CO_2_ can be produced anaerobically [18].

Tissue metabolism also influences the respiratory exchange ratio (RER), i.e., the amount of CO_2_ produced per each mole of O_2_ consumed. Oxidation of lipids releases less CO_2_ (0.7 moles) than oxidation of amino acids (0.84 moles) or carbohydrates (1.0 mole) for 1 mole of consumed O_2_. After reaching anaerobic threshold, RER increases well above 1.0. In analogy, anaerobic metabolism in peripheral tissues can be detected by calculating a surrogate of tissue RER:

(5) $$\begin{array}{c} {\text{RER}}_{\text{sur}}=\frac{{\text{pCO}}_{2}\text{ gap}}{\text{Act}\left(B\right){\text{O}}_{2}-\text{Vct}\left(B\right){\text{O}}_{2}}. \end{array}$$

The value of RER_sur_ > 1.4 (when pCO_2_ gap is in (mmHg) and ctO_2_ in (ml/dL)) was found to be associated with the presence of tissue hypoxia [24]. This is the analogy of the increase of whole-body respiratory quotient when a person exercising on a treadmill overcomes the aerobic threshold. Indeed, RER can be elevated even with normal pCO_2_ gap, if venoarterial difference of oxygen content is very low, as could be the case when significant left-to-right shunting (e.g., at microcirculation level) is accompanying tissue ischaemia.

To summarize, unlike oxygen in the opposite direction, CO_2_ is able to reach the bloodstream regardless of the status of microcirculation. According to the Fick principle, mixed venous-to-arterial CtCO_2_ gap is inversely related to cardiac output and central venous-to-arterial pCO_2_ gap seems to be its acceptable surrogate. In the light of this, it can be hypothesized that even outside the context of sepsis, pCO_2_ gap can be a useful aid in the differential diagnosis of lactic acidosis. In particular, elevated pCO_2_ gap can identify patients who would benefit from fluids and/or other measures aimed at increasing cardiac output. In order to support this hypothesis, we present a series of patients in whom pCO_2_ gap was measured, and the cause of lactic acidosis was conclusively known.

## 2. Methods

In a clinical information system (MetaVision ver. 6, IMD Soft, Israel) that contains data of 5251 patients admitted to 22 bed ICU of the Department of Anaesthesia and Intensive Care of FNKV University Hospital since 2012, we retrospectively searched for patients who had central venous and arterial blood gases measured simultaneously (within 2 min) and also had elevated lactate within 24 hours of paired blood gas measurement. From the list of patients fulfilling these criteria, we selected those where 2 clinicians independently agreed on lactic acidosis being either type A or B, and the diagnosis was either beyond all doubts (e.g., a young fit trauma victim with active bleeding and hemorrhagic shock) or supported by additional evidence (e.g., findings at autopsy). All the rest of the cases were labeled as undetermined.

In those cases, we calculated arterial and venous blood CO_2_ content as

(6) $$\begin{array}{c} {\text{ctCO}}_{2}\left(B\right)=9.286*10^{-3}*{\text{pCO}}_{2}*\text{ctHb}*\left[1+10^{\left(1+10^{\left({\text{pH}}_{\text{ERY}}-{\text{pK}}_{\text{ERY}}\right)}\right)}\right]+{\text{ctCO}}_{2}\left(P\right)*\left(\frac{\text{ctHb}}{21}\right), \end{array}$$

where

(7) $$\begin{array}{c} {\text{ctCO}}_{2}\left(P\right)=0.23*{\text{pCO}}_{2}+{{\text{cHCO}}_{3}}^{-}\left(P\right)\\ {\text{cHCO}}_{3}^{-}\left(P\right)=0.23*{\text{pCO}}_{2}*10^{\left(\text{pH}-{\text{pK}}_{\text{p}}\right)\;},\\ {\text{pK}}_{\text{p}}=6.125-\log\left(1+10^{\left(\text{pH}-8.7\right)}\right),\\ {\text{pH}}_{\text{ERY}}=7.19+0.77*\left(\text{pH}-7.4\right)+0.035*\left(1-{\text{sO}}_{2}\right),\\ {\text{pK}}_{\text{ERY}}=6.125-\log\left(1+10^{\left({\text{pH}}_{\text{ERY}}-7.84-0.06*{\text{sO}}_{2}\right)}\right). \end{array}$$

These equations were from the manual of ABL-800 blood gas machine (Radiometer, Denmark), and indices “ERY” indicate erythrocyte (red blood cell)/.

Oxygen content in arterial blood was calculated as

(8) $$\begin{array}{c} \text{Act}\left(\text{B}\right){\text{O}}_{2}\left(\frac{\text{mL}}{\text{dL}}\right)\;=\;\left[1\text{.}34*\mathrm{Hb}\left(\frac{\text{g}}{\text{dL}}\right){*\mathrm{SaO}}_{2}*0\text{.}01\right]\;+\;\left[\text{0}{\text{.}0225*\mathrm{PaO}}_{2}\left(\text{kPa}\right)\right] \end{array}$$

Oxygen content in venous blood was correspondingly calculated as

(9) $$\begin{array}{c} \text{Vct}\left(\text{B}\right){\text{O}}_{2}\left(\frac{\text{mL}}{\text{dL}}\right)\;=\;\left[1\text{.}34*\mathrm{Hb}\left(\frac{\text{g}}{\text{dL}}\right){*\mathrm{SvO}}_{2}*0\text{.}01\right]\;+\;\left[\text{0}{\text{.}0225*\mathrm{PvO}}_{2}\left(\text{kPa}\right)\right] \end{array}$$

The study was performed in accordance with the Declaration of Helsinki. Because of retrospective and epidemiological nature of the study, informed consent was not required.

## 3. Results

Out of all 5,251 patients in the database, we have found 23 cases with nonseptic patients with elevated lactate and both arterial and venous gases measured. Out of these, there were 6 cases where the diagnosis of either type A or B was beyond any reasonable doubt as independently agreed by 2 clinicians.

### 3.1. Case Series

#### 3.1.1. Example Case 1: Type A Lactacidaemia due to Global Hypoperfusion

A 40-year-old male attempted suicide by jumping out of 3^rd^ floor window. He was intubated at scene, brought in, and diagnosed complex pelvic fracture and compressive fracture of L3. Parenchymatous organs were without signs of injury. After volume resuscitation and blood transfusions, he was haemodynamically stable and remained sedated with plan to operate fractures the next day. In very early hours of the next morning, he suddenly developed signs of haemorrhagic shock with tachycardia 150/min, haemoglobin drop from 129 to 92 g/L, and hypotension with an increase of noradrenalin dose from 0.4 to 1.1 *μ*g·kg^−1^·min^−1^. Lactate at this stage was 1.4 mM (only 90 min later increasing to 3.4 mM), ScvO_2_ 68%, pCO_2_ gap was 1.02 kPa, and RER 1.86. CT scan was repeated and showed a haemoperitoneum and *R*-sided haemothorax due to right-sided diaphragmatic injury that included rupture and bleeding from the teres hepatis ligament. He was classified as having type A lactic acidosis due to haemorrhagic shock.

#### 3.1.2. Example Case 2: Type A Lactacidaemia due to Local Ischaemia

A 53-year-old female, previously fit and well, underwent Whipple's pancreatoduodenectomy due to pancreatic tumour. The operation and the immediate postoperative course were uneventful. She was extubated and haemodynamically stable, passing urine and not requiring vasopressors. Three hours after surgery, she developed severe abdominal pain despite functional epidural analgesia. Her lactate increased to 5.3 mM, ScvO_2_ was 71%, pCO_2_ gap 1.06 kPa, and RER 1.58. She was diagnosed small bowel ischaemia due to occlusion of superior mesenteric artery on angio-CT and underwent relaparotomy and an aortomesenteric bypass operation. She was classified as type A lactic acidosis based on perioperative finding of ischaemic bowel.

#### 3.1.3. Example Case 3: Type A Lactacidaemia due to Combination of Global and Local Hypoperfusion

A 25-year-old man, previously fit and well, completely transsected his brachial artery by a broken glass. He suffered massive blood loss and was found in profound shock by emergency services. He was brought in after having received 2 L of crystalloids and 1 g of tranexamic acid. This patient with evident tissue hypoperfusion due to acute blood loss, with lactate 7.2 mM and ScvO2 58%, had pCO2 gap 1.38 kPa (10 mmHg) and RER of 2.5. In addition, CT scan performed at admission revealed a nonocclusive ischaemia and necrosis of the small bowel that required laparotomy and bowel resection. He was classified as type A lactic acidosis based on perioperative finding of ischaemic bowel.

#### 3.1.4. Example Case 4: Type B Lactacidaemia due to Metformin Overdose

A 71-year-old female with type 2 diabetes was admitted after suicidal attempt committed due to metformin overdose. On admission, she was unconscious, intubated and ventilated, haemodynamically unstable (noradrenaline dose 0.53 *μ*g·kg^−1^·min^−1^), and in profound acidosis (pH 6.58), with lactate 17 mmol/L, SvO2 was 78.7%, pCO2 gap 0.37 kPa (2.8 mmHg), and RER 1.14. She was classified as type B lactic acidosis due to metformin overdose [25] by consensus of clinicians.

#### 3.1.5. Example Case 5: Type B Lactacidaemia due to High Dose Steroids and Betamimetics

A 66-year-old obese female with a history of asthma was hit by a car whilst crossing the road. At scene, she was confused and complaining of shortness of breath. She was intubated and air-lifted to the trauma centre. She was stable and found to have no injuries on the CT scan, and the decision was made to wake her up and extubate. Her pre-extubation lactate was 1.7 mM. Immediately after extubation, she developed severe bronchospasm, which did not respond to inhalatory betamimetics (salbutamol 5 + 5 mg), IV steroids (methylprednisolone 125 mg), and mandated reintubation. Oxygenation was maintained above 93% throughout. Lactate 30 min after intubation, whilst she was sedated and still paralysed, was 7.4 mM, ScvO_2_ 76%, pCO_2_ gap 0.3 kPa, and RER 0.78. Steroids were discontinued for the next few hours, lactate decreased again, and the lady was successfully extubated. She was classified as posteriori by consensus of clinicians as having type B lactic acidosis due to a combined effect of betamimetics [26] and steroids [27].

#### 3.1.6. Example Case 6: Type B Lactacidaemia in Patient with Advanced Multiple Myeloma

A 75-year-old male with known advanced multiple myeloma was referred for sudden-onset paraplegia. He was found to have T8 fracture and underwent an urgent decompressive spinal surgery. Periprocedurally, he also received spinal dose steroids. Blood loss was estimated to 1.6 L and he required perioperative transfusion of blood products, but remained haemodynamically stable. Perioperative blood gas showed lactate 12.8 mM, ScvO_2_ 72%, pCO_2_ gap 0.3 kPa, and RER 0.71. He was referred to ICU after operation. Despite persistent elevation of lactate, he remained stable and was extubated. He was discharged to ward after 5 days, in much improved condition but with lactate still in range of 5–10 mM, a phenomenon that has been described in patients with multiple myeloma [28–30]. He was diagnosed by a posteriori consensus of clinicians as having type B lactic acidosis due to multiple myeloma.

Data from cases of type A and B lactic acidosis are described in Table 1.

### 3.2. Differences between Patients with Type A and B Lactacidaemia

As summarised in Table 1, all patients with typical type A lactic acidosis had pCO_2_ gap well above 0.8 kPa (6 mmHg) including patients who had normal ScvO_2_. On the contrary, all patients with type B lactic acidosis had pCO_2_ gap around 0.4 kPa (3 mmHg). Similar or even better discrimination is obtained when RER is used (Table 1). We found a close correlation between ctCO_2_ gap and pCO_2_ gap (*R*^2^ = 0.71 data not shown), but not so close correlation between ctCO_2_ gap and ctO_2_ gap (*R*^2^ = 0.54, data not shown). Venoarterial lactate difference [31, 32] was in range of −0.3 to +0.2 mM and we have not found any difference between patients with type A and B lactacidaemia.

## 4. Discussion

**Figure 3:**
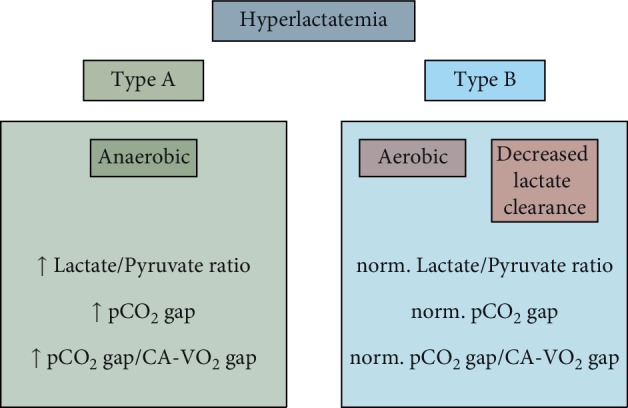
Main physiological features of type A and B hyperlactataemias.

The presented review of physiology and the small case series generate the hypothesis that the use of venous-arterial pCO_2_ gap can be a useful aid in the differential diagnosis of hyperlactataemia. Type A lactic acidosis caused by global or local tissue hypoperfusion with a switch to anaerobic metabolism, whilst type B lactic acidosis is mostly characterized by the absence of tissue hypoxia, and the cause of elevated lactate is either aerobic production or decreased lactate clearance (Figure 3).

Type B lactic acidosis is seen in the heterogeneous group of diseases that are of common occurrence in a typical general ICU [33]. Aerobic lactate production can occur as a paraneoplastic phenomenon (by Warburg effect [34, 35]) or more often as the result of *β*-adrenergic receptor stimulation either during a sympathetic surge (e.g., acute severe asthma, subarachnoidal haemorhage, and pheochromocytoma) or after administration of *β*-receptor agonists (typically *β*_2_-mimetics or adrenaline). Decreased lactate clearance is seen in metformin or ethanol poisoning or in liver failure. Steroids cause hyperlactaemia [36] by a combination of increased aerobic lactate production and decreased lactate clearance [27]. Indeed, many patients would have a combination of type A and B causes of lactic acidosis (e.g., those with septic shock [37, 38], but also many others [39]) (Table 2).

Because the principal difference of type A and B of hyperlactataemia is the presence or absence of tissue hypoxia, the ratio of lactate/pyruvate has been proposed to aid the differential diagnosis of elevated lactate. Due to the ubiquitous presence of cytosolic lactate dehydrogenase lactate/pyruvate ratio reflects the redox situation of cells, i.e., [NADH]/[NAD^+^] ratio, tissue hypoxia would lead to a block of reoxidation of NADH back to NAD^+^. Accumulated NADH would cause leftward shift of equilibrium shown in the following equation:

(10) $$\begin{array}{c} \text{ actate}+{\text{NAD}}^{+}⟷\text{pyruvate}+\text{NADH}. \end{array}$$

Hence, in the presence of any cessation of electron transfer chain (of which by far the most common cause is hypoxia), the lactate/pyruvate ratio increases above normal value of 10 : 1, which is considered a hallmark of type A lactic acidosis [33, 39] In line, type B lactic acidosis would have both lactate and pyruvate increased, with the ration 1 : 10 remaining constant. Nonetheless, the lactate/pyruvate ratio remains of very limited clinical use because currently it is impossible to measure pyruvate concentration by point-of-care techniques, and its laboratory assay is complex and prone to preanalytical errors [39]. Despite technical complexity, the study of lactate/pyruvate ration in critically ill patients with elevated lactate demonstrated that a large proportion of them do not have the biochemical signs of tissue hypoxia and have in general a better prognosis. Indeed, those patients are unlikely to benefit from measures aimed to increase systemic oxygen delivery and more fluids or inotropes may actually cause harm. Unnecessary fluid loading is performed very often in the critically ill (as evidenced in FENICE study [3]), and we can only speculate how often this is triggered by elevated lactate being misinterpreted as a marker of tissue hypoperfusion. Anecdotally, ordering of fluid boluses as automatic response to elevated lactate has been termed “fluid reflex”. We suggest that the role of pCO_2_ gap in detecting tissue hypoperfusion is further studied as a possible marker of tissue hypoperfusion in situations of elevated lactate outside the context of sepsis. We demonstrate in our series of case reports that, in pCO_2_ gap reflected ctCO_2_ gap and was elevated (>1 kPa) in patients with global or local hypoxia, but remained low (<0.5 kPa) in patients with type B lactic acidosis, perhaps due in part to the hyperkinetic circulation that often accompanies diseases associated with type B hyperlactataemia. The pCO_2_ gap cut-off of 0.8 kPa, used in studies on early-goal-directed therapy in septic shock [18, 19] seems to be applicable to our nonseptic cases, too, but indeed, the cut-off with best sensitivity and specificity remains to be determined. Unlike ScvO_2_, the use of pCO_2_ gap is less distorted by the presence of microcirculatory shunts, and hence it should have higher sensitivity to detect tissue hypoperfusion.

Nonetheless, our small observational case series is by no means the proof of the hypothesis outlined above. The next step would be a prospective trial looking at lactate dynamics after a fluid bolus in relation to pCO_2_ gap at baseline in patients with elevated lactate and with cardiac output continuously monitored. Also, much larger series of patients is needed to determine what is the proportion of hyperlactatemic patients in which pCO_2_ gap might be helpful as compared with those with mixed type A + B hyperlactatemias. Lastly, it remains unclear how specific and sensitive pCO_2_ gap is in detecting organ hypoperfusion in patients with mixed etiologies of lactic acidosis.

In conclusion, in this paper, we present a hypothesis that venous-arterial pCO_2_ gap may be a useful aid in the differential diagnosis of elevated lactate in critically ill patients and that it has potential to avoid administration of unnecessary fluids and ionotropics in patients, who have lactate elevated in the absence of tissue hypoperfusion. We demonstrate, for the first time in the literature, that pCO_2_ gap is elevated in nonseptic patients with type A lactic acidosis and normal in type B lactic acidosis.

## Figures and Tables

**Table 1:** Case series of patients with different causes of elevated lactate.

Case	Lac (a) (mmol/L)	SaO_2_ (%)	ScvO_2_ (%)	pCO_2_ gap (kPa)	RER	Type of lactic acidosis and timing of diagnosis	Evidence for classifying hyperlactataemia as either A or B
#1	3.4	99	68	1.02	1.86	A—a posteriori	Peroperative finding
#2	5.3	99	71	1.06	1.58	A—a posteriori	Peroperative finding
#3	7.2	100	58	1.38	2.5	A—a priori	Consensus of clinicians
#4	17.0	99	79	0.37	1.14	B—a priori	Consensus of clinicians
#5	7.4	94	76	0.30	0.78	B—a posteriori	Consensus of clinicians
#6	12.8	99	72	0.30	0.71	B—a posteriori	Consensus of clinicians

Note: Lac a = lactate (arterial); RER = respiratory exchange ratio calculated as per equation (5).

**Table 2:** Overview of causes of elevated lactate.

	Group	Mechanism	Condition/disease	Expected finding
A (increased lactate production)	Low global oxygen delivery leading to excessive anaerobic glycolysis	Severe hypoxia	Any cause (pO_2_ < 4 kPa)	High pCO_2_ gap fluids and ↑ cardiac output are likely to help
		Low O_2_ transport capacity	CO poisoning	
			Severe anaemia	
		Low cardiac output = hypodynamic shock	Low preload (hypovolaemia)	
			Low contractility (cardiogenic)	
			High afterload (obstructive)	
		Normal or high cardiac output, but demand even higher	Strenuous exercise	Fluids and ↑ cardiac output may or may not help
			Shivering or seizures	
	Local ischaemia leading to excessive anaerobic glycolysis	Inflow occlusion	Limb ischaemia	
			Mesenteric ischaemia	
		Decreased perfusion pressure	Compartment syndromes	
		Local ischaemia (Wartburg effect)	Cancer	
	Increased glycolysis in the presence of enough oxygen	Stimulation of muscle and liver glycogenolysis	Beta-2-mimetics	Low pCO_2_ gap fluids and ↑ cardiac output likely to cause harm
			Adrenalin (exogenous or excessive stress)	
			Electrical muscle stimulation [1]	
			Cocaine	
			Theophylline	
		Blocked oxidative phosphorylation (cytopathic hypoxia)	Metformin	
			Cyanide poisoning	
			Propofol-infusion syndrome	
			Methanol	
			Ethylene glycol	
		Production of L- and D-lactate by colon bacteria	Short bowel +	
B (decreased lactate uptake)	Decreased lactate uptake	Liver failure	Acute liver failure	
			Liver ischaemia	
		Failed conversion of pyruvate to AcCoA	Thiamine deficiency	
		Failed conversion of lactate to pyruvate	Alcohol intoxication	

Mixed	Sepsis	Element of hypoxia, aerobic glycolysis, and splanchnic ischaemia		Complex condition
	Propylen glycol poisoning	Mix of D- and L-lactate overproduction and element of oxidative phosphorylation block		

## Data Availability

The deidentified patient's data used to support the findings of this study are *in extenso* provided in the manuscript.

## Abbreviations

Act(B)O_2_: Content of oxygen in whole arterial blood
CaCO_2_: Total carbon dioxide content in whole arterial blood
Central venous blood: Sampled from a central venous catheter (inserted into vena cava superior)
CO: Cardiac output
ct(B)CO_2_ gap: Arterial-to-venous carbon dioxide content difference
ct(B)O_2_ gap: Arterial-to-venous oxygen content difference
CvCO_2_: Total carbon dioxide content in whole venous blood
DO_2_: Oxygen delivery
EGDT: Early goal-directed therapy
Hb: Haemoglobin
MALA: Metformin-associated lactic acidosis
Mixed venous blood: Sampled from a pulmonary artery catheter
pCO_2_ gap: Central venous-to-arterial carbon dioxide partial pressure difference (in the literature ΔPCO_2_ is also used)
RQ: Respiratory quotient
S_v_O_2_: Central venous oxygen saturation
Vct(B)O_2_: Content of oxygen in whole venous blood.
